# Loss of UCHL1 promotes age-related degenerative changes in the enteric nervous system

**DOI:** 10.3389/fnagi.2014.00129

**Published:** 2014-06-19

**Authors:** Josée Coulombe, Prasanna Gamage, Madison T. Gray, Mei Zhang, Matthew Y. Tang, John Woulfe, M. Jill Saffrey, Douglas A. Gray

**Affiliations:** ^1^Centre for Cancer Therapeutics, Ottawa Hospital Research InstituteOttawa, ON, Canada; ^2^Biomedical Research Network, Department of Life, Health and Chemical Sciences, Open UniversityMilton Keynes, UK; ^3^Department of Biochemistry, Microbiology, and Immunology, University of OttawaOttawa, ON, Canada

**Keywords:** ubiquitin, deubiquitinating enzyme, aging, enteric nervous system, glutathione

## Abstract

UCHL1 (ubiquitin carboxyterminal hydrolase 1) is a deubiquitinating enzyme that is particularly abundant in neurons. From studies of a spontaneous mutation arising in a mouse line it is clear that loss of function of UCHL1 generates profound degenerative changes in the central nervous system, and it is likely that a proteolytic deficit contributes to the pathology. Here these effects were found to be recapitulated in mice in which the *Uchl1* gene had been inactivated by homologous recombination. In addition to the previously documented neuropathology associated with loss of UCHL1 function, axonal swellings were detected in the striatum. In agreement with previously reported findings the loss of UCHL1 function was accompanied by perturbations in ubiquitin pools, but glutathione levels were also significantly depleted in the brains of the knockout mice, suggesting that oxidative defense mechanisms may be doubly compromised. To determine if, in addition to its role in the central nervous system, UCHL1 function is also required for homeostasis of the enteric nervous system the gastrointestinal tract was analyzed in UCHL1 knockout mice. The mice displayed functional changes and morphological changes in gut neurons that preceded degenerative changes in the brain. The changes were qualitatively and quantitatively similar to those observed in wild type mice of much greater age, and strongly resemble changes reported for elderly humans. UCHL1 knockout mice should therefore serve as a useful model of gut aging.

## Introduction

UCHL1 is a member of the ubiquitin carboxyterminal hydrolases, a subgroup of the deubiquitinating enzymes. UCHL1 was first detected as an abundant protein in the human brain by two-dimensional gel analysis. It was estimated that PGP9.5 (as the protein was originally designated) constituted 1–2% of soluble brain protein. Based on its abundance and restricted expression UCHL1 has been used extensively as a pan-neuronal marker (later studies revealed the expression of UCHL1 is not limited to neurons). The discovery of UCHL1 and much of the later literature on neuronal and non-neuronal expression has been admirably reviewed (Day and Thompson, 2010).

The structure and biochemical activities of UCHL1 can be considered unusual. Like UCHL3 (a closely related but ubiquitously expressed enzyme) UCHL1 has a “knotted” structure which when projected on a plane displays five crossings (Virnau et al., 2011). The catalytic domain of UCHL1 differs from that of UCHL3 by the presence of a loop covering the active site (Das et al., 2006). In the absence of any conformational change this loop would obstruct access of any ubiquitinated substrate larger than a small peptide, which would explain the restriction of UCHL1 to such substrates *in vitro* (Larsen et al., 1998). Two possibilities (which are not mutually exclusive) exist for the *in vivo* activity of the enzyme: UCHL1 cleaves ubiquitin from an as yet unidentified bulky substrate following conformational change, or the substrates are genuinely small. Proposed roles for UCHL1 are the processing of the ubiquitin proprotein by cleavage of the short C terminal peptide, and salvage of ubiquitin from remnants of proteasomal degradation proteins. Both activities have been demonstrated *in vitro* (Larsen et al., 1998). Both activities would be essential for maintenance of ubiquitin pools and proteolytic efficiency in neurons, and it has been demonstrated that in the absence of UCHL1 neuronal ubiquitin pools are significantly depleted (Osaka et al., 2003). In addition to cleaving ubiquitin-peptide bonds UCHL1 very efficiently cleaves ubiquitin-thioester bonds (Larsen et al., 1998), suggesting an intriguing possibility for the homeostatic function of the enzyme. Glutathione is a small cysteine-containing tripeptide of established relevance to neuronal homeostasis (Shaw, 1998). In a 1983 publication Irwin Rose and Jessie Warms documented the thioesterase activity of UCHL1 (Rose and Warms, 1983) and speculated that this activity may serve to salvage ubiquitin from spontaneous thioesters formed with glutathione (a speculation repeated in Rose's Nobel prize lecture of 1994). An obvious mechanism for formation of these conjugates would be transthioesterification with glutathione supplanting the E2 conjugating enzymes or E3 ligases of the ubiquitin cascade. To our knowledge this activity has not been documented *in vivo*. To determine if UCHL1 provides this function *in vivo* we assayed ubiquitin and glutathione levels in the nervous system of our newly generated line of UCHL1 knockout mice and their wild type littermates.

Much of what is known of UCHL1 function *in vivo* has been determined through analysis of GAD (gracile axonal dystrophy) mice, in which spontaneous deletion of exons 7 and 8 removes essential domains of the enzyme (Saigoh et al., 1999). GAD mice show progressive loss of hind limb function associated with axonal swellings in the gracile tract of the spinal cord. Neurons with the long processes are most affected, and there is a “dying back” from distal regions of the axon toward the cell body (Miura et al., 1993). Because rare polymorphisms in the human *UCHL1* gene (also known as *PARK5*) have been associated with altered risk for Parkinson's disease (Wintermeyer et al., 2000; Maraganore et al., 2004) we examined dopaminergic neuronal projections in the striatum of UCHL1 null mice. In Parkinson's disease and other neurodegenerative conditions the dysfunction seen in CNS neurons similarly affects those in the periphery, and it is therefore reasonable to assume that the absence of UCHL1 would also affect neuronal populations outside the CNS. The enteric nervous system (ENS) is comprised of a vast and highly organized network of neurons and glial cells orchestrating gastrointestinal motility, secretion, and absorption. UCHL1 is abundantly expressed in the two major ganglionated plexuses that comprise the ENS (Grundy and Brookes, 2012), but has not been studied in the context of ENS homeostasis. The gastrointestinal function of UCHL1 null mice and wild type littermate controls was therefore analyzed functionally and the ENS of these animals was examined for signs of neuropathology.

## Materials and methods

### Generation of UCHL1 knockout mice

**Figure 1:**
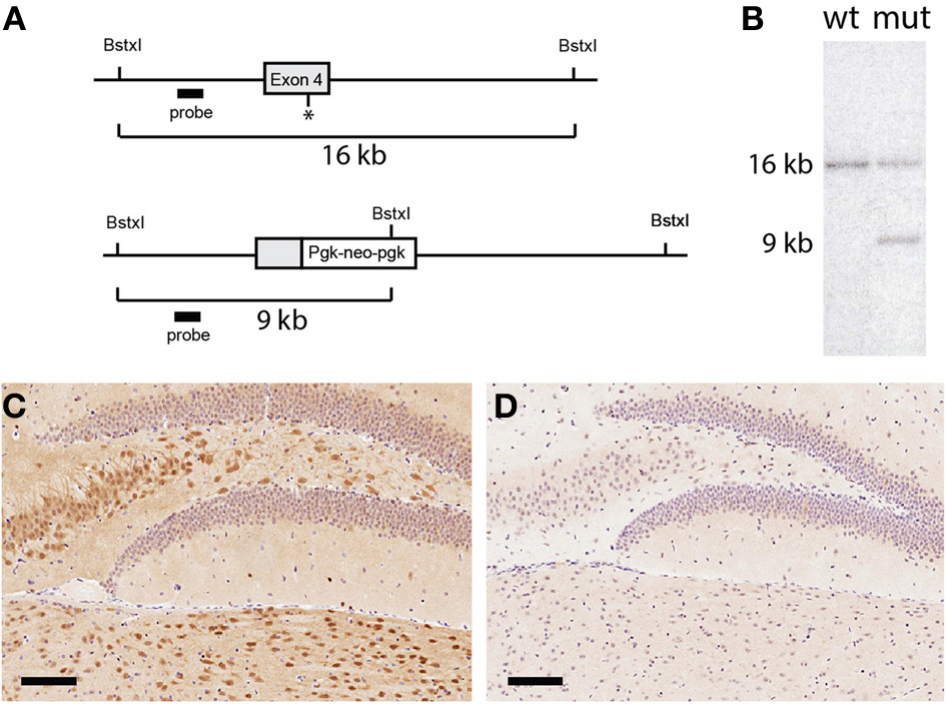
**Generation of UCHL1 null mice. (A)** Schematic of the mutational strategy. A region spanning exon 4 of the mouse UCHL1 gene was chosen for gene targeting. Through homologous recombination a portion of the exon including the catalytically essential cysteine at position 90 (asterisk) was replaced with a Pgk-neo-pgk selectable marker. **(B)** Confirmation of gene targeting. A probe generated from an intronic fragment detected the expected 16 kilobase fragment in Southern blotting analysis of wild type genomic DNA digested with the restriction endonuclease BstxI. Introduction of an additional cleavage site in the selectable marker generated a smaller 9 kilobase DNA fragment in genomic DNA from a G418-resistant embryonic stem cell clone, confirming that one copy of the UCHL1 gene had been targeted. This clone was used to generate the UCHL1 knockout line. **(C)** UCHL1 immunoreactivity in the dentate gyrus of a wild type mouse. In wild type mice UCHL1 immunoreactivity could be detected throughout the central nervous system; staining of the dentate gyrus was representative of other regions. **(D)** Absence of UCHL1 immunreactivity in the dentate gyrus of a UCHL1 null mouse. This mouse was a littermate of the mouse in **(C)** (both were products of a heterozygous cross). Scale bars = 100 microns.

The mouse UCHL1 gene was cloned from a 129sv phage genomic library using methods that have been previously described (Di Fruscio et al., 1998). A 16 kb region of the gene spanning the fourth exon was used to generate the targeting vector pDG250 (represented schematically in Figure 1), in which the exon was replaced with the selectable marker pgk-neo-pgk phosphoglycerate kinase promoter/neomycin phosphotransferase/phosphoglycerate kinase 3′ untranslated region. The pDG250 vector was linearized using NotI and transfected in R1 ES cells (the gift of Andras Nagy, University of Toronto) via electroporation. Clones resistant to G418 (Geneticin, Life Technologies, Burlington, Ontario, Canada) were screened for the desired recombination event by Southern blotting of BstxI digested DNA using a genomic probe derived from the third intron. Chimeras were produced by the aggregation of properly recombined clones with CD-1 morulas and germ line transmission was established. The 129 line was established by crossing the chimera with a 129S1/SvImJ female and is maintained by intercrossing heterozygous offspring. A second line in the C57BL/6J genetic background was established by backcrossing to a C57BL/6J (The Jackson Laboratory, Bar Harbor, Maine, USA) for 10 generations before intercrossing the heterozygous offspring.

### Western blotting

Proteins from freshly dissected tissues were solublized in protein lysis buffer (20 mM Tris-HCl pH 7.5, 150 mM NaCl, 1% Nonidet P-40 (Sigma, St. Louis, Missouri, USA), 0.5 mM EDTA, 20% glycerol) containing the following protease and phosphates inhibitors: 1 mM phenylmethylsulfonyl fluoride, 5 μg/mL leupeptin (Sigma, St. Louis, Missouri, USA), 2 μg/mL aprotinin (Sigma, St. Louis, Missouri, USA), 200 μM sodium Fluoride (NaF) and 200 μM sodium pyrophosphate (NaPPi). The cells were incubated on ice for 30 min and then centrifuged at 14 000 rpm for 20 min at 4°C to pellet cellular debris. The soluble fractions were recovered and the protein concentration was determined using the Bradford protein assay (Bio-Rad Laboratories Inc., Mississauga, Ontario, Canada). Twenty or thirty micro gram of cytoplasmic extracts were then resolved on a two-phase SDS-polyacrylamide gel (15 and 8%) and electroblotted onto a hybond C nitrocellulose membrane (Amersham Pharmacia Biotech, Baie D'Urfé, Québec, Canada). The membranes were stained with Ponceau S (Sigma, St. Louis, Missouri, USA) prior to western blotting with the appropriate antibody to ensure the complete transfer of the proteins. The primary anti-ubiquitin antibody (DAKO Diagnostics Canada Inc., Burlington, Ontario, Canada) and secondary anti-rabbit antibody were diluted in 5% skim milk in TBST (10 mM Tris-HCl (pH7.6), 150 mM NaCl and 1% Tween-20) for 1 h at room temperature. The membranes were washed 3 times with TBST prior to incubation with the appropriate secondary antibody. The proteins were detected by a horseradish peroxidase method and SuperSignal West Pico Chemiluminescent Substrate reagents (Pierce, Rockford, IL, USA) and were visualized using a GeneGnome instrument (Syngene, Frederick, Maryland, USA).

### Immunohistochemistry

Brains from age-matched wild type and UCHL1 knockout mice were excised and fixed in 10% phosphate-buffered formalin for 2 days at room temperature. Tissues were paraffin-embedded and sectioned coronally using a microtome at a thickness of 5 μm. Deparaffinized sections were heated in a solution of 10 mM sodium citrate (pH 6.0) in 700 W microwave for 10 min. Endogenous peroxidase activity was blocked by incubating in methanol containing 3% hydrogen peroxide for 20 min. Sections were washed with 0.1 M PBS (pH 7.4) and incubated for 30 min with 1.5% normal rabbit, goat, or horse serum (Santa Cruz Biotechnologies Inc, SC, CA, USA) as appropriate to block non-specific binding. Sections were then incubated overnight at 4°C with the antibody specific for UCHL1 (EMD Millipore, Darmstadt, Germany) or ubiquitin (DAKO Diagnostics Canada Inc., Burlington, Ontario, Canada). Sections of the striatum were stained with an anti-tyrosine hydroxylase antibody (Immunostar, Hudson, WI, USA) at a dilution of 1:4000. Reaction products were visualized with the ABC system (DAKO Diagnostics Canada Inc., Burlington, Ontario, Canada).

### Glutathione analysis

For analysis of glutathione levels *in vivo* 129sv UCHL1-KO mice were sacrificed at 3 months of age. The animals were dissected and the hindbrains and/or transverse colon collected. The total glutathione content of the organs was evaluated colorimetrically using a gluthathione assay kit (Cayman Chemical, Ann Arbor Michigan, USA) as per the method of Baker (Baker et al., 1990) and of Tietze (Tietze, 1969). Briefly, samples were homogenized in MES (2-(N-morpholino)ethanesulphonic acid) buffer and proteins as well as protein complexes were precipitated out of solution with metaphosphoric acid. With the proteins precipitated, remaining thiols in the sample were presumed to be glutathione. After the sample pH was re-established with triethanolamine, DTNB (5,5′-dithio-*bis*-2-(nitrobenzoic acid), Ellman's reagent) was added and conjugates to reduced glutathione (GSH) produced yellow colored TNB (5-thio-2-nitrobenzoic acid). Glutathione reductase present in solution ensured all oxidized glutathione (GSSG) was reduced to GSH, making the test a measure of the total free glutathione (i.e., not bound to protein) in the sample. Samples were analyzed on an MRX microplate reader (Dynex Technologies, Chantilly Virginia, USA) using the instrument's Revelation software (version 3.04).

For analysis of glutathione in cultured cells NIH3T3 cells (ATCC, Manassas Virginia, USA) were plated at 8,000 per well in an opaque 96-well plate. After 24 h, the cells were transfected with 0.5 μg of either plasmid pDG268 (a wild-type ubiquitin/eGFP fusion Tsirigotis et al., 2001b) or pcDNA control using the GeneJuice transfection reagent (EMD Biosciences, Darmstadt, Germany). At 48 h, LDN-91946 (3-Amino-2-benzoyl-6-oxo-6,7-dihydrothieno[2,3-b]pyridine-5-carboxylic acid), a selective, uncompetitive inhibitor of UCHL1 (Mermerian et al., 2007), was dissolved in DMSO and added to cells at 25 μM and buthionine sulfoximine (BSO) to other wells at 200 μM. At 72 h, cell culture media was removed, the cells were washed in PBS and the reduced glutathione (GSH) content of these cells was measured using a GSH-Glo Glutathione Assay (Promega, Madison WI, USA) on a Glo-Max luminometer (Promega, Madison Wisconsin, USA).

### Whole mount analysis

Wholemounts of the myenteric plexus in the ileum of null, heterozygous and wildtype littermates were prepared as previously described for the mouse colon (Gamage et al., 2013). Ileum samples were removed and placed for 20 min in PBS containing the calcium channel blocker nicardipine hydrochloride (10^−6^ mol L^−1^) to ensure maximal (and hence comparable) smooth muscle relaxation. Specimens were then opened longitudinally along the mesentery and circumference (C) and length (L) were measured. Specimens were stretched maximally (but without tearing of tissue), pinned on Sylgard-lined plates and fixed for 24 h at room temperature in modified Zamboni's fixative (2% paraformaldehyde, 0.2% picric acid in 0.1 molL^−1^ sodium phosphate buffer, pH 7.4). Samples were then washed in 100% dimethyl sulphoxide (DMSO) for 3 × 10 min followed by 3 × 10 min washes in PBS. The circumference (C′) and the length (L′) were then re-measured. The muscularis externa was separated from underlying layers under a dissecting microscope, the circular muscle layer was removed and wholemounts of the longitudinal muscle with myenteric plexus attached were prepared. Wholemounts were then incubated in PBS pH7.4 containing lysine 1 mg/ml, 0.01% bovine serum albumin, 0.05% sodium azide (antibody diluting solution, ABDS) and 0.5% Triton × 100 (to aid antibody penetration), for 2 h at room temperature, followed by 10% normal goat serum (Dako) in ABDS with 0.1% Triton × 100 for 2 h at room temperature to reduce non-specific binding of antibodies. Wholemounts were incubated overnight (18 h) at room temperature in rabbit anti PGP 9.5 (Ultraclone, RA95101. 1:) or anti-nNOS (ImmunoStar, Inc; 24287, 1:1000) antibodies, washed in PBS (3 × 10 min), then incubated in secondary antibodies (Invitrogen, Goat anti Rabbit Alexa 488, 1:200) in darkness at room temperature for 2 h. Wholemounts were then washed in PBS (3 × 10 min) and mounted with Citifluor (Agar Scientific Limited).

### Quantification of nNOS-IR neuronal numbers

To obtain quantitative data on nNOS–IR neuronal numbers, 7 random confocal Z stacks (1024 × 1024 pixels; optical section 2 μm) per specimen were captured using a confocal laser scanning microscope (Leica DM6000 CS) equipped with a 20X objective, which defined an area of (775 × 775 μ m^2^) on the specimen. Aerial images for each of the 7 random areas were obtained by overlaying optical sections of the respective Z stack. Quantitative analysis of images was performed by an experimenter blind to the tissue from which the images were taken. For each of the 7 images obtained per wholemount, the total number of neurons labeled by nNOS antibody was counted using Image Pro Plus software (Media Cybernetics Inc, Bethesda, MD, USA). nNOS-IR neurons counted on all 7 images of a wholemount were pooled to obtain the total neuronal count for that wholemount. Only neurons lying within the optical field and those intersecting the top and right hand edges were counted. The number of neurons per unit area varies with the stretch of the wholemount. Therefore, a correction factor for stretch was employed. The stretch correction factor was calculated [C × L]/[C′ × L′] for each wholemount and then the actual unstretched area (A) of the wholemount represented by 7 images was calculated [stretch correction factor × stretched area sampled = stretch correction factor × 4.20 mm^2^]. To obtain the stretch-corrected number of neurons/mm^2^, each of the pooled neuronal counts obtained above were divided by the actual unstretched area (A) of the respective wholemount.

### Transit time analysis

Transit time was measured using methodology described by Kuo et al. (2010). Mice were single caged 1 day prior to the experiment. At a recorded time mice were gavaged with 0.2 ml of carmine dye (6% w/v: Sigma-Aldrich Canada Co., Oakville, Ontario, Canada) in 0.5% methyl cellulose (Sigma-Aldrich Canada Co., Oakville, Ontario, Canada). The gavage needle was dipped in a solution of 1 g/ml sucrose prior to introducing it to the mouse (for a calming effect). The cage bedding was changed to a white piece of Iso Pad (Omni Bio Resources, Cherry Hill, New Jersey, USA) to facilitate the observation of colored feces. The time at which the first colored pellet was seen was recorded and the transit time was calculated as the interval between gavage and appearance of the first colored stool. Data were analyzed for significance by Student's *t*-test.

## Results

### Inactivation of the *Uchl1* gene results in neurodegeneration

Genomic DNA spanning the fourth exon of the UCHL1 gene (encoding an essential cysteine at position 90) was isolated from a 129sv genomic library and was used to generate a targeting vector in which part of the exon was replaced with a selectable marker (Figure 1A). An embryonic stem cell clone was identified in which the expected homologous recombination event had occurred (Figure 1B). This clone was used to generate a knockout line in the 129sv genetic background. Whereas the UCHL1 protein was abundant in wild type brains it was undetectable in sections from mice homozygous for the targeted gene analyzed by immunohistochemistry (Figures 1C,D). UCHL1 protein could not be detected in lysates from the spinal cords of homozygous mice (**Figure 3A**), nor could the *Uchl1* transcript could be detected by reverse transcriptase/polymerase chain reaction (not shown). It is probable that the presence of the selectable marker destabilizes the *Uchl1* gene transcript, but with no RNA or protein product detectable we conclude that the targeting event has generated a true null mutation. By crossing our original line with C57BL/6J mice for more than 10 generations a second UCHL1 knockout line in the C57BL/6J background was produced. At the molecular level these lines are indistinguishable.

Both lines of UCHL1 null mice manifest neurodegenerative phenotypes strongly reminiscent of the GAD spontaneous mutant (Yamazaki et al., 1988). Mice homozygous for the null mutation can be identified as early as 6 weeks of age by the failure to spread their hindlimbs when suspended by the tail (in this and other behavioral assays heterozygous mice are indistinguishable from wild type animals). At various ages the UCHL1 mice have comparable performance on a rotating rod (data not shown) to that reported for GAD mice (Yamazaki et al., 1992). With regard to weight all three genotypes are indistinguishable at 12 weeks of age at which point null mice plateau and are sacrificed when the differential between their weight and healthy littermates reaches 20%.

**Figure 2:**
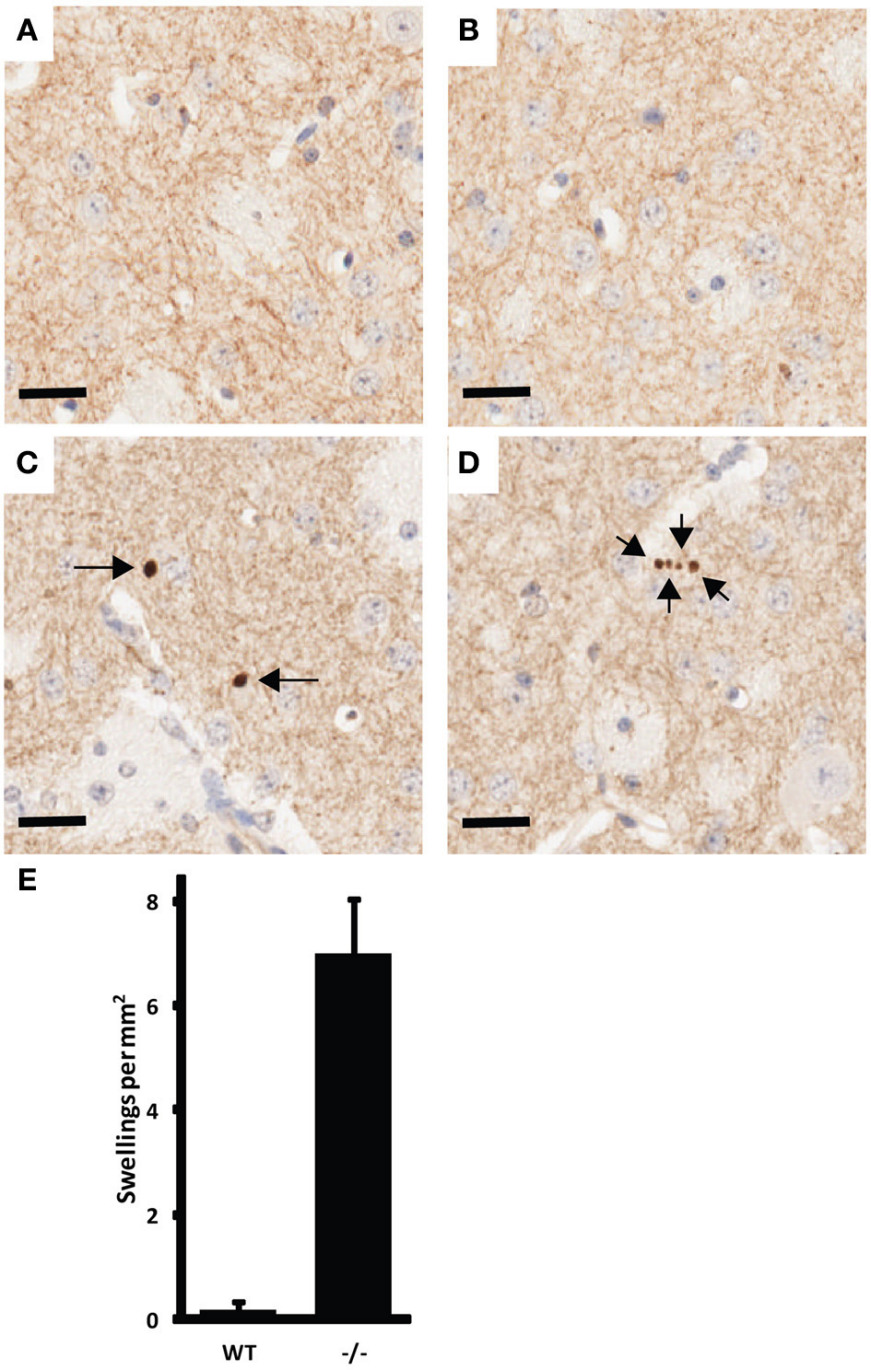
**Example of CNS pathology associated with UCHL1 ablation**. Tyrosine hydroxlase (TH) positive axonal swellings in the striatum of 3-month old UCH-L1 mice. **(A,B)** are representative images from wild type mice. **(C,D)** are representative images from UCHL1 null littermates. Arrows indicate the position of axonal swellings. Scale bars = 20 microns. **(E)** Number of TH positive axonal swellings present in 5 high-power fields (captured with 40x objective) per mouse (*n* = 3 for wild type; *n* = 4 for UCHL1 null). Error bars represent the standard error of the mean. The difference between wild type and null mice was found to be significant (*p* < 0.005).

Examination of histological sections of the CNS of UCHL1 null mice revealed pathology similar to that reported for GAD mice, including axonal swellings in the gracile nucleus and in the cerebellar peduncles (not shown). Our examination was extended to the striatum, which contains processes from dopaminergic neurons in the substantia nigra and could therefore be analyzed to detect a “dying back” phenomenon. Multiple axonal swellings that stained positive for tyrosine hydroxylase (TH) were detected in coronal sections of striatum from 3-month old UCHL1 null mice show (Figure 2). These swellings were almost non-existent in wild type littermate controls. The difference was found to be significant (*p* < 0.005).

### UCHL1 is required for maintenance of ubiquitin and glutathione pools

**Figure 3:**
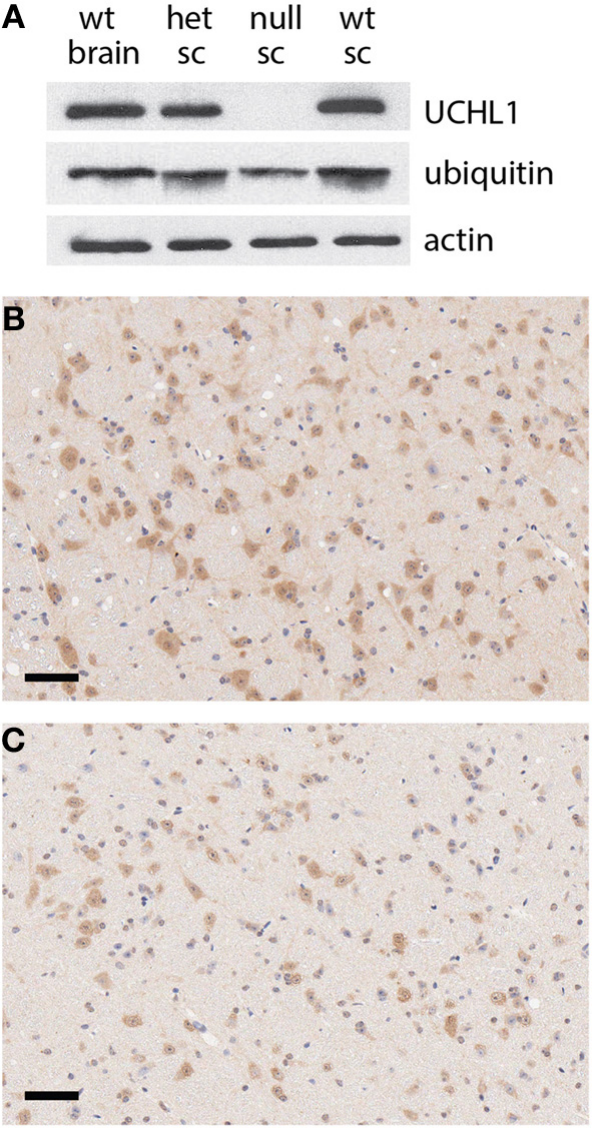
**Reduction in ubiquitin pools in UCHL1 null mice. (A)** Western blot analysis of protein levels in the brain of a wild type mouse and the spinal cords (sc) of heterozygous (het), UCHL1 null, and wild type littermates from a heterozygous cross. No UCHL1 protein was detectable in the lysate of the null animal, coincident with a reduced level of monomeric ubiquitin. Beta actin levels appeared equivalent, consistent with equal loading. **(B)** Ubiquitin immunoreactivity in the gigantocellular reticular nucleus of a wild type mouse. **(C)** Reduced ubiquitin immunoreactivity in the gigantocellular reticular nucleus of a UCHL1 null mouse a littermate of the wild type mouse in panel **(B)**. Scale bars = 100 microns.

**Figure 4:**
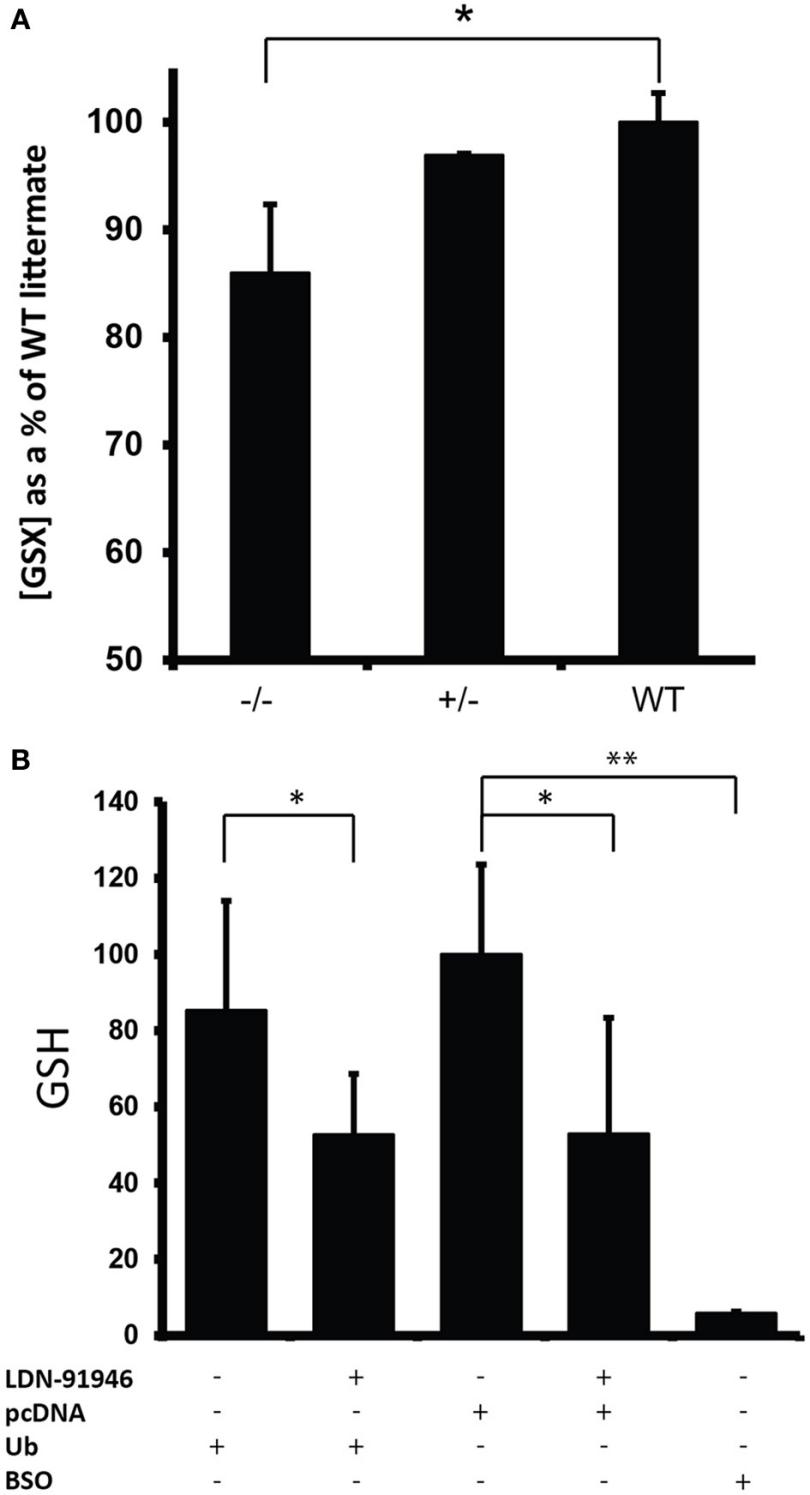
**Reduction in glutathione levels in the absence of UCHL1 activity. (A)** Free glutathione levels (GSH/GSSG) in mouse hindbrain. Shown are the average level of glutathione for each genotype (*n* = 8 for UCHL1 null, *n* = 2 for heterozygous, *n* = 6 for wild type) normalized to the average levels of wild type littermates (set at 100%). Error bars represent the standard deviation. ^*^p < 0.005. **(B)** Reduced glutathione (GSH) levels in NIH3T3 cells treated with LDN-91946. A pharmacological inhibitor of UCHL1. Shown are the average GSH levels normalized to the average of control cells transfected with the empty pcDNA vector (set at 100%). Error bars represent the standard deviation. ^*^p < 0.05 (*Ub* = 0.017, pcDNA = 0.025) ^**^p < 0.001 (BSO = 0.000538).

Through a non-catalytic mechanism UCHL1 binds to and stabilizes monomeric ubiquitin (Osaka et al., 2003). In light of this activity it is to be expected that the total ablation of UCHL1 protein would result in a reduction in monomeric ubiquitin pools, a prediction borne out by western blot analysis of spinal cord lysates (Figure 3A). These data were in agreement with immunohistochemical analysis of total ubiquitin in various regions of the brain, including the gigantocellular nucleus (Figures 3B,C). An activity that has been postulated for UCHL1 but not previously demonstrated *in vivo* is salvage of glutathione from the ubiquitin-glutathione thioester adducts known to be promiscuously generated in cells (Rose and Warms, 1983). The null mice provide an appropriate platform for testing the hypothesis that UCHL1 provides the salvage function. Free glutathione (oxidized and reduced) was found to be markedly decreased (86%, *p* < 0.002) in the brains of mice null for UCHL1 as compared to wild type littermates (Figure 4A). No significant difference was found between heterozygous and wild type mice. To account for differences in total protein content and brain size, Bradford assays were performed on the samples prior to glutathione quantification. Hindbrain protein content was found to be comparable across genotypes (data not shown). Glutathione assays were also performed on gut tissues, but no significant difference was detected between knockout mice and wild type littermates (not shown). Given the small contribution of ENS cells to the mass of the GI tract (relative to smooth muscle and secretory cells) it is likely that any difference in ENS glutathione would be obscured by non-neuronal sources of the tripeptide and rendered undetectable. Future studies will attempt to explore the effects of UCHL1 ablation on the glutathione pools of primary cultures of enteric neurons isolated from the gut, but at present our data are limited to the CNS and effects on ENS glutathione pools seem plausible but are entirely speculative.

The availability of a pharmacological inhibitor of UCHL1 (Mermerian et al., 2007) allowed us to corroborate the glutathione effect in cultured cells. NIH3T3 cells transfected with an empty pcDNA control plasmid followed by treatment with uncompetitive UCHL1 inhibitor LDN-91946 showed a significant decrease (*p* < 0.05) in reduced glutathione (GSH) when compared to cells similarly transfected but without inhibitor treatment (Figure 4B). To determine the limiting entity in glutathione-ubiquitin thioester formation ubiquitin levels were augmented by transfecting cells with pDG268, an expression vector encoding a ubiquitin-eGFP. In transfected cells the ubiquitin-eGFP fusion protein is co-translationally processed by deubiquitinating enzymes to produce functional monomeric ubiquitin (Tsirigotis et al., 2001a). Expression of the eGFP component was confirmed by fluorescence microscopy of transfected cells (not shown). No significant difference was detected in the GSH levels of cells transfected with pDG268 vs. cells transfected with the empty pcDNA plasmid (Figure 4B), indicating that pre-existing ubiquitin pools did not limit thioester formation. Buthionine sulfoximine (BSO) inhibits glutamate-cysteine ligase, the initial enzyme in glutathione biosynthesis. It therefore provides an effective control for glutathione measurement. With the addition of BSO we observed near total depletion of the glutathione pool (*p* < 0.001).

### Morphological abnormalities in the ENS of *Uchl1* knockout mice resembling those of aged animals

**Figure 5:**
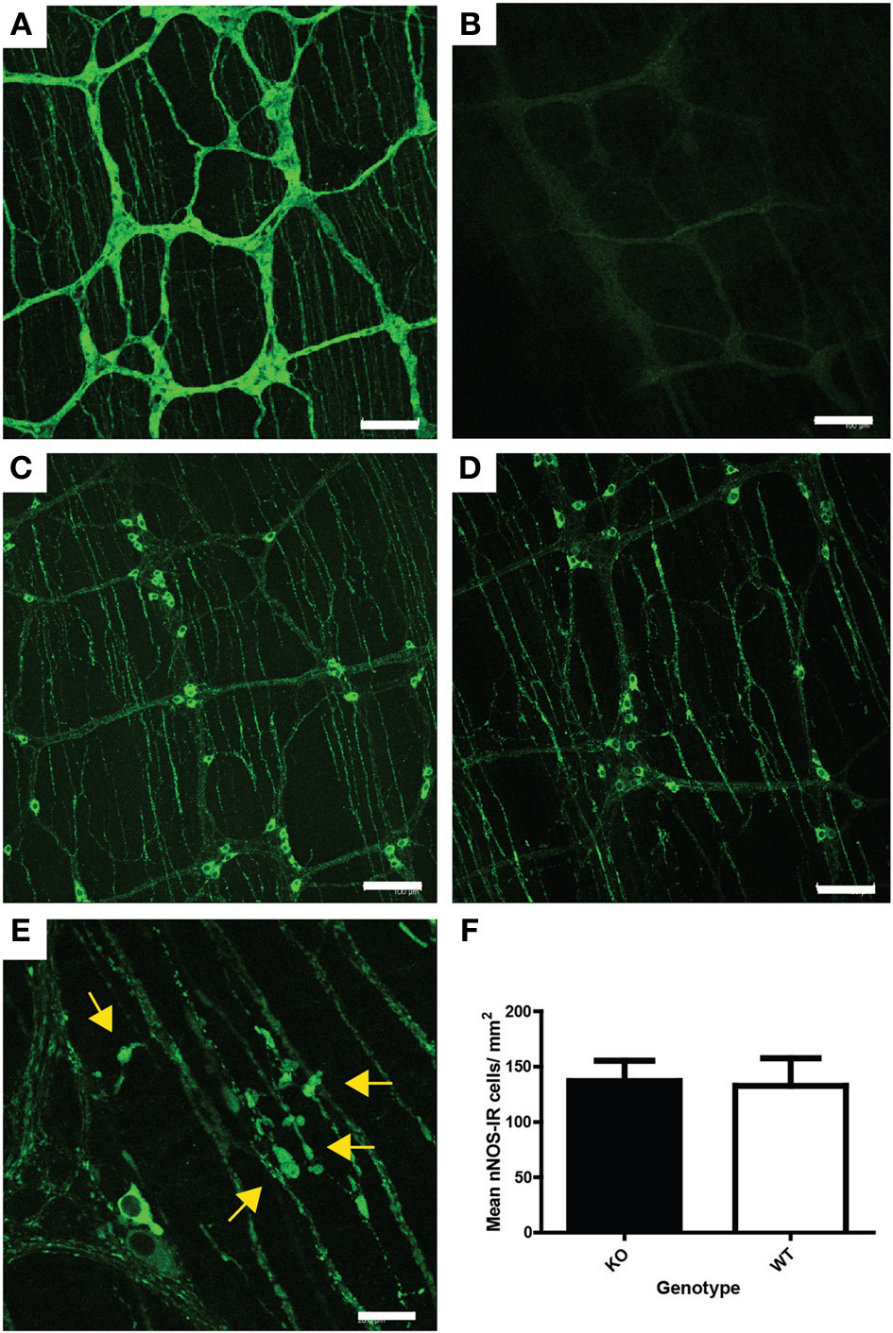
**ENS pathology associated with UCHL1 ablation. (A)** PGP 9.5 (UCHL1) immunoreactivity in wildtype mouse ileum. Myenteric ganglia linked by nerve fiber bundles are clearly visible. Immunoreactive neuronal cell bodies and nerve fibers can be seen in the ganglia and thinner parallel nerve fiber bundles in the tertiary plexus (which innervates the longitudinal muscle layer) are also present. Scale bar = 100 microns **(B)** Lack of PGP 9.5 immunoreactivity in null mouse ileum. Scale bar = 100 microns **(C)** nNOS immunoreactive neurons and nerve fibers in the myenteric ganglia and fibers in the tertiary plexus of wildtype mouse ileum. Scale = 100 microns **(D)** nNOS immunoreactive neurons and nerve fibers in the myenteric ganglia and fibers in the tertiary plexus of null mouse ileum. Fibers in the tertiary plexus and ganglia appear larger and brighter than in those in Figure 5D. Scale = 100 microns **(E)** Higher magnification showing swollen nNOS immunoreactive fibers (yellow arrows) in the tertiary plexus and myenteric ganglion of a null mouse ileum. Scale bar = 20 microns **(F)** Density of nNOS immunoreactive neurons in the myenteric plexus of null (KO) and wildtype mouse ileum. No significant difference in neuronal numbers was detected by Mann–Whitney test. Raw cell counts were corrected for varied stretch of the wholemounts.

The ENS of *Uchl1* knockout, heterozygous and wildtype littermates was studied in wholemount preparations of the myenteric plexus of the ileum by immunofluorescence techniques. Wholemounts were immunolabeled for UCHL1 using anti PGP 9.5 antiserum (UCHL1 is also known as protein gene product 9.5, or PGP 9.5, see 1). Previous studies have shown that PGP 9.5 immunoreactivity is detected in almost all myenteric neuronal cell bodies and in nerve fibers, and it has been widely used as a pan-neuronal marker in the ENS. As expected, no immunoreactivity was detected in the ENS of knockout mice (compare Figures 5A,B).

To study the effect of *UCHL1* knockout on the morphology of myenteric neurons, one of the major subpopulations of myenteric neurons, nitrergic neurons, was chosen for analysis. We chose to concentrate our studies on the ileum because the ENS is better characterized in that region than in other gut regions, including other regions of the small intestine. Nitrergic neurons constitute approximately 26% of the total myenteric population in the mouse ileum (Qu et al., 2008), and they play a key role in smooth muscle relaxation, and hence in gastrointestinal motility. Nitrergic neurons in wholemount preparations were immunolabeled using an antiserum raised against nNOS (neuronal nitric oxide synthase). Immunolabeling of nNOS in knockout and in wild-type littermates is shown in Figures 5C,D. No change in the appearance of neuronal cell bodies was observed, but numerous swollen process were seen in the knockout animals (Figure 5E). These swellings resemble those seen in the ENS of ageing mice (Gamage et al., 2013). There was no change in the number of nNOS-IR myenteric neurons in either the knockout or heterozygous mice, when compared to wildtype controls (Figure 5F). Neither was there a large scale atrophy; measurement of ileum circumference prior to whole mount preparation failed to reveal difference in mice of the various genotypes (not shown).

### Evidence for reduced GI function in *Uchl1* knockout mice and in aged control mice

**Figure 6:**
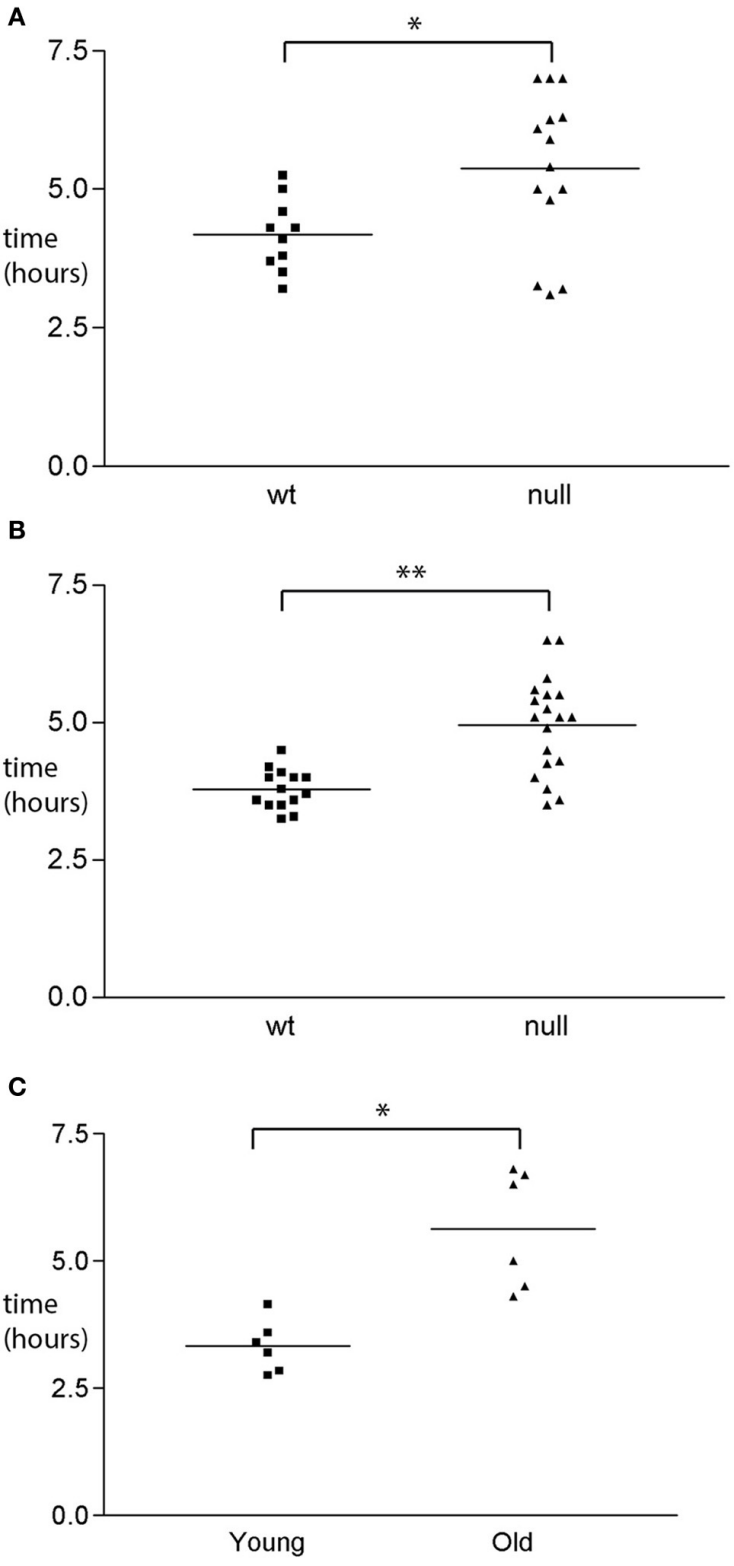
**GI functional changes associated with UCHL1 ablation. (A)** Transit time analysis of wild type and UCHL1 null male mice of the C57BL/6 genetic background. Mice were 8–12 weeks of age (average 10 weeks). Each symbol represents the transit time for one mouse. The horizontal line represents the mean transit time value. ^*^p < 0.05 **(B)** Transit time analysis of wild type and UCHL1 null female mice of the C57BL/6 genetic background. Mice were 8–12 weeks of age (average 10 weeks). Each symbol represents the transit time for one mouse. The horizontal line represents the mean transit time value. ^**^p < 0.0001 **(C)** Transit time analysis of young (8 week) and older (26 month) mice of the C57Bl/ICRFAt1 genetic background Each symbol represents the transit time for one mouse. The horizontal line represents the mean transit time value. ^*^p < 0.05.

The structural abnormalities detected in the ENS of mice null for UCHL1 suggested that gastrointestinal function may be altered in these mice. Dysfunction of the myenteric plexus would be expected to compromise the smooth muscle relaxation and/or contraction orchestrated within the ENS, culminating in a motility deficit. To determine if this was the case we measured the transit time for a bolus of colored dye introduced into the stomach to be expelled by defecation. Transit times were measured for male and female cohorts of homozygous null mice and wild type littermates in both the 129Sv and C57BL/6J genetic backgrounds. Data are shown for the C57BL/6J cohorts (Figures 6A,B), but were similar for the 129Sv mice. We observed an increase of approximately 40% in the transit time of UCHL1 mice of both sexes, a difference that was found to be statistically significant. To determine how this genetically induced deficit in gastrointestinal motility compared to “natural” age-related decline we performed identical transit time analyses in cohorts of young and older C57BL/ICRFAt1 mice (2 month and 26 months of age respectively, roughly corresponding 8 and 80 years of age in the human Turnbull et al., 2003) obtained from the aged rodent colony at Newcastle University, Newcastle upon Tyne, United Kingdom. The survival of mice in this colony at 26 months of age is approximately 50% (see Supplementary Materials). There was a remarkable concordance in our findings (Figure 6C): genetic ablation of UCHL1 results in reduced intestinal motility equivalent to that caused by 2 years of aging in the mouse (an interval that would equate to decades of human aging).

## Discussion

The finding that UCHL1 null mice have a progressive neurodegenerative phenotype culminating in hindlimb paralysis was not unexpected; indeed our intention in eliminating a portion of the catalytic domain of UCHL1 was to ensure that any protein produced from the gene would be inactive, as is the case for the spontaneous deletion of the seventh and eighth UCHL1 exons in GAD mouse line (Saigoh et al., 1999). Similarly, our finding that ubiquitin pools are reduced in the absence of the UCHL1 protein is in good agreement with previous findings (Osaka et al., 2003) and suggests that perturbation of ubiquitin-mediated proteolysis in the knockout mice could contribute to their pathology. If so, the situation could be greatly exacerbated by a diminution of glutathione levels in the nervous system. Glutathione provides a critical anti-oxidant defence to neurons, as evidenced by the apoptotic cell death of primary neurons in which glutathione levels have been reduced pharmacologically (Andersen et al., 1996) or by knockdown of the rate-limiting enzyme for biosynthesis (Diaz-Hernandez et al., 2005). Fibroblasts from mice engineered to be deficient in glutathione by knockout of glutamate-cysteine ligase are hypersensitive to oxidative damage; mice null for the catalytic subunit of the enzyme die *in utero* (Dalton et al., 2000). Based on the existing literature it is unlikely that the 15% reduction in glutathione levels we have documented in the brains of UCHL1 knockout mice could alone account for the extensive, ultimately fatal neurodegeneration in our mice (or in the GAD mouse line). Rather we speculate that partial depletion of glutathione may compromise anti-oxidative defences sufficiently to task the ubiquitin-proteasome system with the degradation of an added burden of oxidatively damaged protein. Over the course of time the added burden may be particularly problematic for neurons with relentless metabolic demands and limited proteolytic reserves. Motor neurons with extremely long axonal processes may fall into this category (perhaps due to the requirement for active proteolysis at distant synaptic sites, and the incessant demands for transport of biosynthetic and degradative components to these sites). Another potentially vulnerable neuronal population is dopaminergic neurons within the substantia nigra. There is evidence that dopamine metabolism imposes an inherent level of oxidative stress that can at least in part explain why the substantia nigra is a major locus of Parkinson's disease (Tsang and Chung, 2009). Swollen striatal axons are found in both the MPTP (Turner et al., 1988) and α-synuclein overexpression (Decressac et al., 2011) models of Parkinson's disease. Nigrostriatal fibers are not the most abundant striatal afferents but TH-positive axons originate specifically from the substantia nigra. Nigral degeneration is the hallmark feature of PD and glutathione depletion has been implicated as a triggering event in PD pathogenesis (Perry et al., 1982; Jenner et al., 1992; Nakamura et al., 1997). Nigral dopaminergic neurons have been shown *in vivo* to be more sensitive to glutathione depletion than non-dopaminergic neurons (Garrido et al., 2011).

Mechanistically we believe that the best explanation for the partial glutathione depletion in UCHL1 null mice is afforded by the salvage pathway (Rose and Warms, 1983). In the absence of this activity the fate of glutathione would be that of ubiquitin itself, which is known to turn over through proteosomal degradation (Shabek and Ciechanover, 2010). Remaining glutathione-ubiquitin conjugates would at a minimum be functionally inert (the thioester bond blocking the residues through which both components normally form covalent bonds to substrates), but the possibility also exists for dominant negative interference with components of anti-oxidative and/or ubiquitin-mediated pathways.

Gastrointestinal disorders are among the problems that plague the elderly, and there has been some effort to determine the extent to which deterioration of the ENS contributes to the loss of gastrointestinal function. Much of this work has been done in model systems, most notably the guinea-pig, the rat, and the mouse. A seemingly simple but fundamental question is whether neurons are lost from the ENS as the organism ages, but simple answers have not been forthcoming. There is considerable variability in the figures reported for age-related neuronal cell death in the ENS of these three species, and even amongst strains of the same species (Saffrey, 2013). Whereas figures as high as 50% have been reported for aged Sprague-Dawley rats (Cowen et al., 2000) it would appear that in the C57BL/6 mouse no neuronal loss occurs in the myenteric plexus of the colon at ages up to 2 years (Gamage et al., 2013). The chief finding in the aged mouse is the accumulation of swollen fibers and varicosities within ganglia and in the fiber bundles that connect ganglia (Gamage et al., 2013). These abnormalities are very similar to the swellings detected in the colonic myenteric plexus of UCHL1 knockout mice (Figure 5). Even in optimal conditions it would be reasonable to assume that metabolically active ENS neurons would be faced with oxidative damage, and there is evidence that levels of reactive oxygen species increase with age in the neurons of the myenteric plexus (Thrasivoulou et al., 2006). The data presented herein demonstrate that genetic ablation of UCHL1 results in reduced pools of monomeric ubiquitin and glutathione (Figures 3, 4, respectively). A reduction in the efficiency of protein turnover and in the chief anti-oxidative defence of ENS neurons may underlie their propensity to develop age-related pathological features precociously.

Within the adult ENS there exist stem cells and/or glia that can be differentiated into neurons *in vitro* (Joseph et al., 2011; Laranjeira et al., 2011). Studies in the adult rat provided no evidence for neurogenesis *in vivo*, and at present there is no reason to believe that damaged, dysfunctional, or dead neurons in the digestive tract are routinely replaced (discussed in Saffrey, 2013). It therefore seems likely that the neurons in the ENS of a centenarian have persisted for 100 years, attesting to their robustness in a fortunate subset of individuals. For other elderly individuals the evidence for gastrointestinal decline is unfortunately inescapable: the elderly are disproportionately represented amongst patients reporting gastrointestinal distress (Wade and Cowen, 2004). Transit time analysis have been performed in human subjects (similar to the studies reported herein, but utilizing radioisotopes rather than dye tracers), and an increase of very similar magnitude was reported for aging humans (Madsen and Graff, 2004) as we have reported for aging or UCHL1 null mice. Even in younger individuals the ENS may be damaged by parasitic infection (Iantorno et al., 2007) or by anticancer therapies (Wafai et al., 2013) or pharmacological interventions. With no prospect of replacing aged ENS neurons it would appear that the preservation of neurons is of paramount importance, but we still know relatively little about potential promoters of ENS neuronal longevity or even of the molecular mechanisms that can compromise such longevity. UCHL1 levels are known to be lower in replaceable CNS neurons of mice and songbirds (Lombardino et al., 2005); the data presented herein suggest that UCHL1 is essential for homeostasis of irreplaceable neurons in the ENS. By providing an accelerated and therefore tractable model of ENS decline, UCHL1-deficient mice should provide insights into ENS aging, and may provide a platform for the assessment of therapeutic interventions to preserve ENS function.

## Author contributions

Josée Coulombe and Prasanna Gamage performed most of the research, with additional contributions from Madison T. Gray, Mei Zhang, and Matthew Y. Tang. John Woulfe and M. Jill Saffrey provided neuropathological analysis of CNS and ENS tissues respectively. Douglas A. Gray designed the project and wrote the manuscript with contributions from Josée Coulombe, Madison T. Gray, John Woulfe, and M. Jill Saffrey.

### Conflict of interest statement

The authors declare that the research was conducted in the absence of any commercial or financial relationships that could be construed as a potential conflict of interest.
