# Supplementary material for: Loss of UCHL1 promotes age-related degenerative changes in the enteric nervous system
Source: Front Aging Neurosci. 2014 Jun 19;6:129. doi: 10.3389/fnagi.2014.00129 (PMC4063237; doi:10.3389/fnagi.2014.00129)
Supplement: Supplementary file 1 [file Presentation1.PDF]

Supplemental Figure. Survival data for the Newcastle C57Bl/ICRFAt1mouse colony. Actual data are represented by solid lines. Dashed lines represent simulated curves of best fit. For both sexes the 50% survival point corresponds to approximately 30 months of age. Data courtesy of Adele Kitching, Newcastle University, Newcastle upon Tyne, United Kingdom.

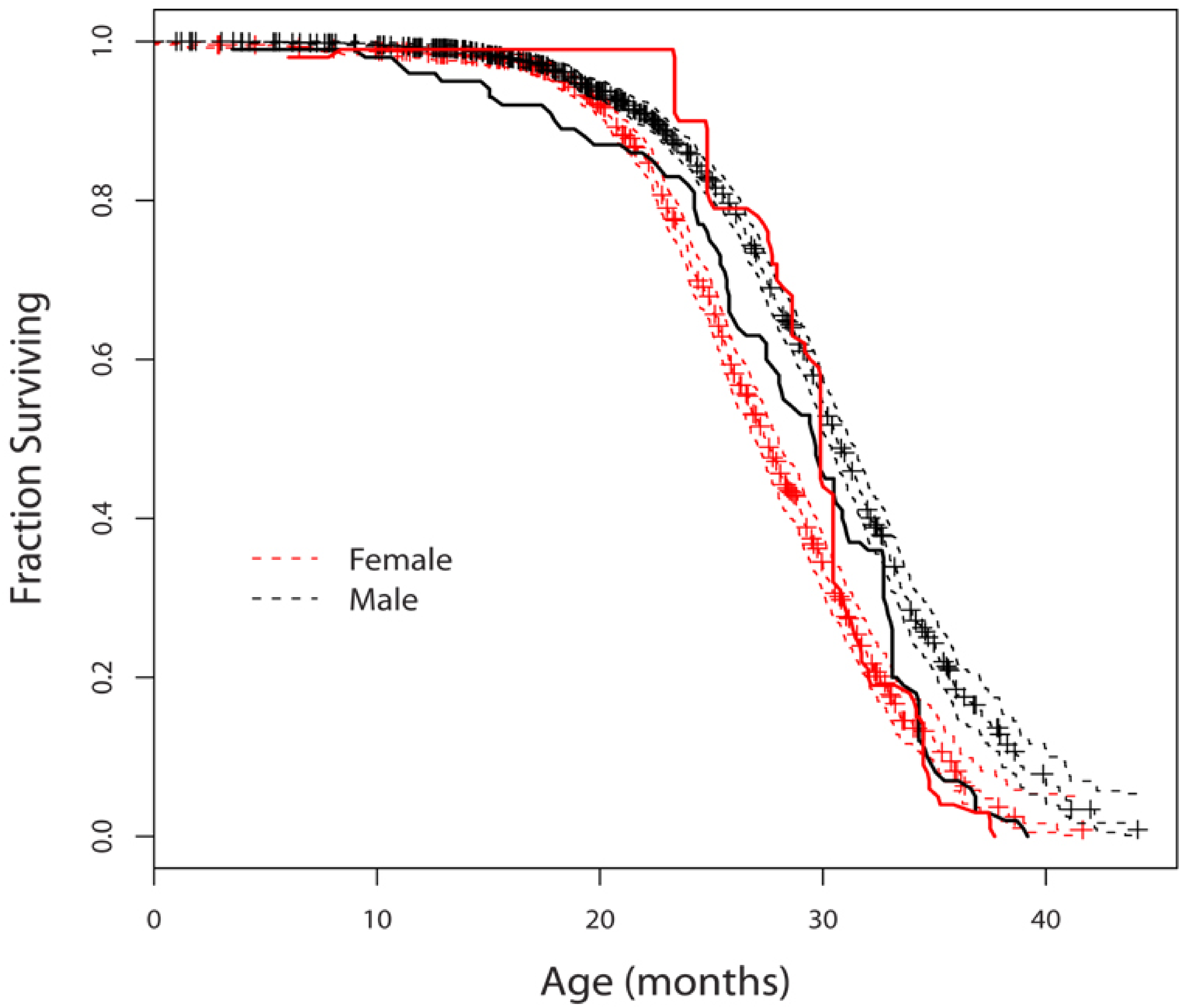

Fraction Surviving
1.0
0.8
0.6
0.4
0.2
0.0
Female
Male
0
10
20
30
40
Age (months)
